# Symptomatic early coronary graft failure in bypass surgery patients: incidence, predictors and clinical impact

**DOI:** 10.1007/s12471-024-01926-z

**Published:** 2025-01-20

**Authors:** Martijn J. H. van Oort, Ibtihal Al Amri, Arend de Weger, Madelien V. Regeer, J. Wouter Jukema, Bart J. A. Mertens, Jose M. Montero-Cabezas

**Affiliations:** 1 Department of Cardiology, Leiden University Medical Centre, Leiden, The Netherlands; 2 Department of Thoracic Surgery, Leiden University Medical Centre, Leiden, The Netherlands; 3 Netherlands Heart Institute, Utrecht, The Netherlands; 4 Department of Biomedical Data Sciences, Leiden University Medical Centre, Leiden, The Netherlands

**Keywords:** Coronary artery bypass graft surgery, Coronary angiography, Symptomatic early coronary graft failure

## Abstract

**Objectives:**

Coronary graft failure (CGF) may occur early after coronary bypass graft surgery (CABG). The study aimed to identify clinical and perioperative risk factors and to evaluate the long-term clinical impact of symptomatic early CGF.

**Methods:**

Patients who underwent clinically indicated coronary angiography (CAG) prior to post-CABG discharge between 2012 and 2022 were included. Symptomatic early CGF was defined as a dysfunctional coronary graft, evaluated on clinically indicated CAG, caused by stenosis of the proximal or distal anastomosis or bypass conduit, bypass occlusion, thrombosis, reduced flow (TIMI < 1) and kinking/tenting. Patients were divided into symptomatic early CGF and non-early CGF groups. Kaplan-Meier and multivariate analysis estimated cumulative survival free of major adverse cardiovascular events (MACE: death, myocardial infarction and revascularisation) up to 5 years’ follow-up and identified predictors of symptomatic early CGF.

**Results:**

A total of 92 patients (79% male, 66.1 ± 10 years old) were included, of whom 55 (59.8%) had symptomatic early CGF. Baseline characteristics, surgical parameters and post-surgical parameters potentially indicative of ischaemia were comparable between groups. Patients with symptomatic early CGF had a significantly lower MACE rate over a median follow-up period of 33 months (*p* = 0.023). Venous graft integration (*p* = 0.005), Y‑graft configuration (*p* = 0.002) and prolonged inotropic support (*p* = 0.032) were associated with symptomatic early CGF.

**Conclusions:**

Symptomatic early CGF was observed in the majority of post-CABG patients undergoing clinically indicated CAG prior to discharge. Patients with symptomatic early CGF exhibited higher MACE rates over a median follow-up period of 33 months. Venous graft integration, Y‑graft configuration and prolonged use of inotropic agents were associated with symptomatic early CGF. However, these clinical findings should be interpreted with caution.

**Supplementary Information:**

The online version of this article (10.1007/s12471-024-01926-z) contains supplementary material, which is available to authorized users.

## What’s new?

- This study provides an angiographic definition of symptomatic early coronary graft failure (CGF).
- Symptomatic early CGF was observed in a majority (60%) of post-coronary artery bypass graft patients undergoing clinically indicated coronary angiography before discharge.
- Symptomatic early CGF patients had a higher risk of major adverse cardiovascular events over a median follow-up period of 33 months.
- Venous graft integration, Y‑graft configuration and prolonged inotropic support were associated with symptomatic early CGF.

## Introduction

Due to rapid advancements in percutaneous cardiac interventions, the complexity of cardiac surgery is rising [1]. In addition, patients undergoing cardiac surgery tend to be older and have more comorbidities, such as diabetes, congestive heart failure and previous percutaneous coronary intervention (PCI), leading to an older and sicker population undergoing surgery [1, 2]. Consequently, the risk of perioperative myocardial injury and infarction (PMI) is increasing [3]. One cause of PMI is coronary graft failure (CGF), which may occur before hospital discharge after coronary bypass graft surgery (CABG) and is associated with adverse clinical outcomes [4–8]. Adequate identification and treatment of early CGF is therefore of utmost importance. This can, however, be challenging, especially shortly after CABG, as traditional indicators of myocardial injury such as angina, dyspnoea, electrocardiographic (ECG) changes, haemodynamic instability or cardiac biomarker elevation (troponin T (TnT) and creatinine kinase (CK)) can occur without myocardial ischaemia [9]. Coronary angiography (CAG) is the gold standard for diagnosing early CGF and guiding revascularisation to prevent further myocardial ischaemia and complications [10]. However, performing CAG poses risks of complications, while clear guidelines are lacking on which patients should undergo early CAG. Additionally, a clear definition of early CGF is missing. This study aimed to propose a definition of symptomatic early CGF based on CAG evaluation, to identify clinical and perioperative risk factors and to define its clinical impact.

## Patients and methods

### Population and data collection

This retrospective observational study included all CABG patients (> 18 years) who underwent unplanned, clinically indicated CAG prior to post-surgery discharge between 2012 and 2022 at the Leiden University Medical Centre (Leiden, The Netherlands). The study encompassed all CABG procedures, including off-pump surgery, as well as CABG with concomitant procedures. CAG evaluation was performed to identify early CGF based on prespecified angiographic criteria. To compare clinical and perioperative variables potentially indicative of symptomatic early CGF, patients were divided into early CGF and non-early CGF groups.

Demographic, clinical, angiographic, echocardiographic, ECG and procedural data were prospectively collected in the departmental information system (EPD-Vision; Leiden, The Netherlands). When available, clinical variables were evaluated at three distinct time points: pre-surgery, immediately post-surgery (perioperative) and 12 h post-surgery, to assess differences potentially indicative of ischaemia. Follow-up data were recorded through review of medical records and retrieval of survival status through municipal civil registries, with data collection closing in May 2024. Patients without data on post-CABG CAG were excluded. The Ethical Committee of the Leiden University Medical Centre (2022-08; 09-03-2022) approved the evaluation of clinically collected data and waived the need for patient written informed consent.

### Definitions and coronary angiographic analysis

All postoperative CAGs were clinically indicated based on clinical criteria evaluated by the treating physician and discussed within a multidisciplinary team, including a cardiothoracic surgeon and an interventional cardiologist. Coronary and bypass angiograms were independently reviewed by two experienced interventional cardiologists and an experienced cardio-thoracic surgeon. Discrepancies were resolved and consensus was achieved through discussion within the team.

Symptomatic early CGF was defined as a dysfunctional coronary graft resulting from any of the following findings: stenosis of proximal or distal anastomosis of the bypass or the Y‑anastomosis, stenosis of the bypass conduit, bypass occlusion, bypass thrombosis, reduced bypass flow (TIMI < 1) and kinking/tenting of the bypass.

Haemodynamic status was evaluated based on the need for inotropic, vasopressive or mechanical support, which were considered prolonged from 12 h after CABG.

### Laboratory evaluation

TnT (μg/l) and CK (U/l) were routinely monitored from hospitalisation until 12 h post-surgery. ∆TnT or ∆CK were calculated as the difference between immediate and 12‑h post-surgical levels.

### Echocardiographic evaluation

Transthoracic echocardiograms (TTEs) were retrospectively analysed by an experienced imaging cardiologist using standard grey-scale images from apical four-chamber, two-chamber and long-axis views using Q‑analysis (EchoPAC version 111.0.0; GE Vingmed, https://www.norwayhealthtech.com/member/ge-vingmed-ultrasound-as/). Left ventricular (LV) ejection fraction was measured using the biplane Simpson’s method and defined as good (> 52% for males, > 54% for females), mildly reduced (41–51% for males, 41–53% for females), moderately reduced (30–40% for males and females) or poor (< 30% for both). When available, postoperative echocardiograms were compared to preoperative echocardiograms to detect new regional wall motion abnormalities (RWMA), described as hypokinetic or akinetic [11].

### Study endpoints

Primary endpoints were clinical and perioperative risk factors of symptomatic early CGF and its clinical impact in terms of major adverse cardiovascular events (MACE: death, myocardial infarction or revascularisation) up to 5 years’ follow-up.

### Statistical methods

Continuous variables are presented as mean ± standard deviation or median with interquartile range (25th–75th percentile), as appropriate. Differences between unpaired continuous variables were assessed with the unpaired *t*-test if normally distributed, and with the Mann-Whitney U test if not normally distributed. Categorical variables were reported as frequencies and percentages and were analysed using the χ2 or Fisher exact test. Kaplan-Meier analysis estimated cumulative survival free of MACE up to 5 years, with group comparisons using the log-rank test. Associations between clinical and perioperative characteristics with symptomatic early CGF were investigated with univariable and multivariable logistic regression. Variables considered relevant or that demonstrated a *p*-value less than 0.2 in the univariate analysis were included in the multivariate analysis. Statistical analysis was performed with SPSS for Windows version 25.0 (IBM, Armonk, NY, USA). All tests were two-sided, and a *p*-value of < 0.05 was considered statistically significant.

## Results

### Patients and angiographic findings

Between 2012 and 2022, 4355 patients underwent CABG, with 94 (2.2%) receiving clinically indicated CAG before discharge. CAG was unsuccessful in 2 patients, resulting in 92 patients (79.3% male, 66 ± 10 years old) being included in the study.

**Fig. 1:**
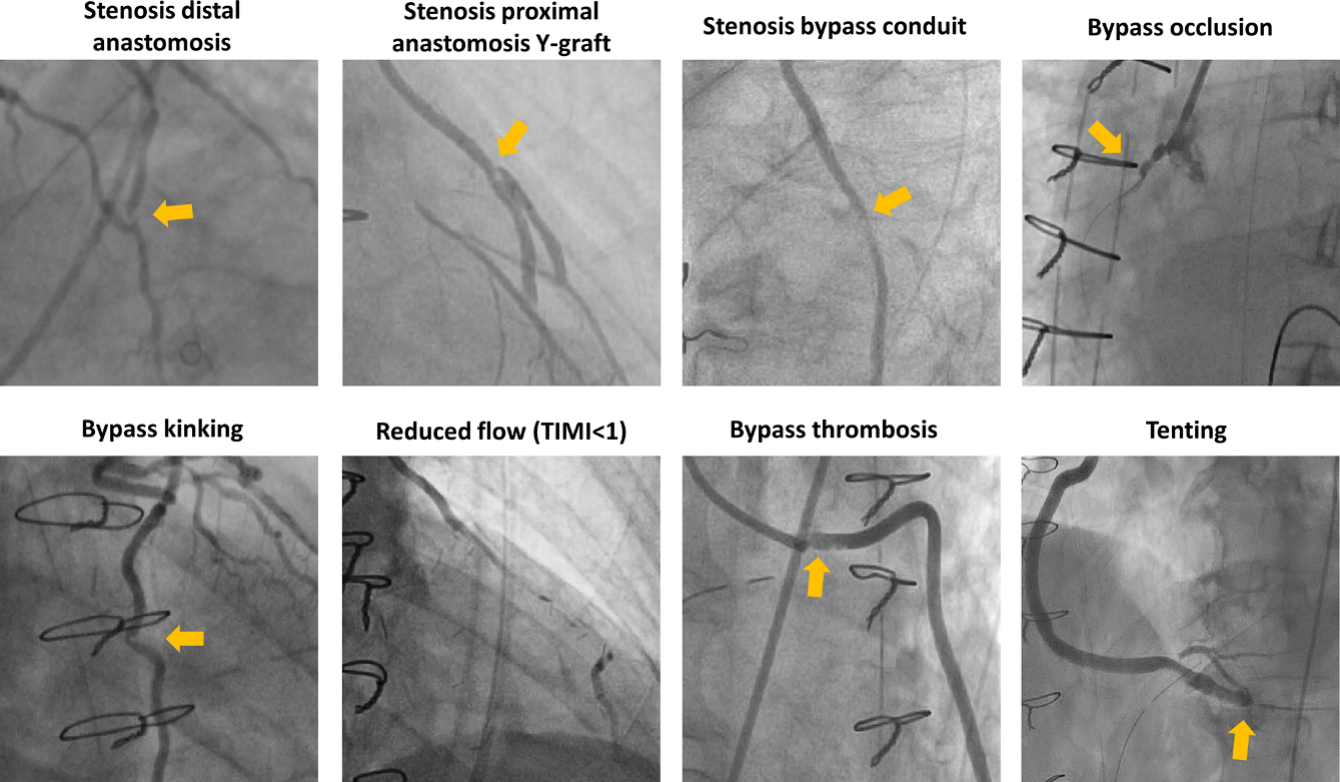
Causes of symptomatic early coronary graft failure in patients undergoing clinically indicated in-hospital coronary angiography after coronary artery bypass graft surgery

Symptomatic early CGF was identified in 55 patients (59.8%), with a total of 81 findings. The most frequent finding was stenosis of the distal anastomosis (*n* = 27, 29.3%), followed by occlusion (*n* = 18, 19.6%), kinking (*n* = 13, 14.1%), stenosis of the bypass conduit (*n* = 9, 9.8%), reduced bypass flow (*n* = 7, 7.6%), stenosis at the level of the Y‑graft anastomosis (*n* = 3, 3.3%), bypass thrombosis (*n* = 2, 2.2%) and tenting due to a too short graft (*n* = 2, 2.2%) (Fig. 1; Tab. 1). Among the Y‑grafts, 12 patients (46.2%) had problems located at the distal anastomosis, with problems predominantly affecting vessels revascularising the anterior and/or lateral territory (*n* = 18, 69.2%).

**Table 1:** In-hospital coronary angiography findings and treatment approaches

	Overall	PCI	Re-CABG	Conservative
*Early graft failure*				
Stenosis of Y‑graft anastomosis, *n* (%)	3 (3.3)	2 (67.7)	0	1 (33.3)
Stenosis of distal anastomosis, *n* (%)	27 (29.3)	15 (55.6)	3 (11.1)	10 (37)
Stenosis of bypass conduit, *n* (%)	9 (9.8)	8 (88.9)	2 (22.2)	1 (11.1)
Reduced bypass flow, *n* (%)	7 (7.6)	2 (28.6)	1 (14.3)	4 (57.1)
Bypass occlusion, *n* (%)	18 (19.6)	10 (55.6)	4 (22.2)	5 (27.8)
Bypass thrombosis	2 (2.2)	1 (50)	1 (50)	0
Kinking of bypass, *n* (%)	13 (14.1)	5 (41.7)	5 (41.7)	4 (33.3)
Tenting of bypass, *n* (%)	2 (2.2)	0	0	2 (100)
*No early coronary graft failure*				
Native vessel thrombosis, *n* (%)	3 (3.3)	3 (100)	0	0
New stenosis of native vessel, *n* (%)	2 (2.2)	2 (100)	0	0
No complete revascularisation after CABG, *n* (%)	2 (2.2)	2 (100)	0	0

Percentages in the PCI, Re-CABG and Conservative columns represent the proportion of each treatment modality out of the total findings for each type of graft failure. In the Overall column, percentages reflect the proportion of each finding within the entire patient cohort *CABG* coronary artery bypass graft, *PCI* percutaneous coronary intervention

### Baseline characteristics and procedural parameters

Baseline characteristics were comparable across both groups (Tab. 2). Most patients had three-vessel disease (*n* = 56, 60.9%), good baseline LV function (*n* = 57, 62.0%), and underwent elective surgery (*n* = 60, 65.2%) mostly after non-ST-elevation myocardial infarction (*n* = 25, 27.2%) and with a median EuroSCORE II of 1.9 (1.2–4.0).

**Table 2:** Baseline characteristics

	Overall (*N* = 92)	No early graft failure (*n* = 37)	Early graft failure (*n* = 55)	*p*-value
Age, years	66.1 ± 10.0	65.6 ± 10.4	66.4 ± 9.8	0.692
Male, *n* (%)	73 (79.3)	31(83.8)	42 (76.4)	0.389
*Primary CAG findings*				
– 1 VD, *n* (%)	12 (13.0)	6 (16.2)	6 (10.9)	0.459
– 2 VD, *n* (%)	24 (26.1)	12 (32.4)	12 (21.8)	0.256
– 3 VD, *n* (%)	56 (60.9)	19 (51.4)	37 (67.3)	0.125
– Left main, *n* (%)	14 (15.2)	6 (16.2)	8 (14.5)	0.827
BMI, g/l^2^	27.1 ± 4.5	26.0 ± 4.0	27.8 ± 4.7	0.057
Diabetes mellitus, *n* (%)	26 (28.3)	10 (27.0)	16 (29.1)	0.829
Hypertension, *n* (%)	57 (62.0)	22 (59.5)	35 (63.6)	0.686
Hypercholesterolaemia, *n* (%)	45 (48.9)	15 (40.5)	30 (54.5)	0.419
History of smoking, *n* (%)	44 (47.8)	17 (45.9)	27 (49.1)	0.767
*Preoperative LV function*				0.788
– Good, *n* (%)	57 (62.0)	23 (62.2)	34 (61.8)	
– Mild reduction, *n* (%)	21 (22.8)	10 (27.0)	11 (20.0)	
– Moderate reduction, *n* (%)	4 (4.3)	1 (2.7)	3 (5.5)	
– Poor, *n* (%)	5 (5.4)	1 (2.7)	4 (7.3)	
*EuroSCORE II*	1.9 (1.2–4.0)	2.0 (1.1–2.9)	1.7 (1.2–4.6)	0.777
*Clinical presentation*				0.314
– Stable angina, *n* (%)	20 (21.7)	5 (13.5)	15 (27.3)	0.117
– Unstable angina, *n* (%)	22 (23.9)	9 (24.3)	13 (23.6)	0.940
– NSTEMI, *n* (%)	25 (27.2)	14 (37.8)	11 (20)	0.059
– STEMI, *n* (%)	9 (9.8)	3 (8.1)	6 (10.9)	0.736
– Other, *n* (%)	16 (17.4)	6 (16.2)	10 (18.2)	0.807
*Urgency of surgery*				0.636
– Elective, *n* (%)	60 (65.2)	23 (62.2)	37 (67.3)	
– Urgent, *n* (%)	27 (29.3)	11 (29.7)	16 (29.1)	
– Emergency, *n* (%)	5 (5.4)	3 (8.1)	2 (3.6)	

*BMI* body mass index, *CAG* coronary angiography, *LV* left ventricular, *VD* vessel disease, *NSTEMI* non-ST-segment elevation myocardial infarction, *STEMI* ST-segment elevation myocardial infarction

Procedural parameters were also mostly similar (Tab. 3). The left internal mammary artery (LIMA) alone was used in 9 cases (9.8%), while both LIMA and right internal mammary artery (RIMA) were used in 26 (28.3%). Among these, 5 (5.4%) were in situ and 21 (22.8%) had a Y-graft configuration. For arterial plus venous grafts, LIMA and saphenous vein graft (SVG) were used in 45 cases (48.9%) and LIMA, RIMA and SVG in 5 (5.4%). Of these, LIMA-SVG(Y) was used in 4 (4.3%) cases and a LIMA-RIMA(Y) and SVG in 1 case (1.1%). Concomitant surgical interventions were performed in 32 patients (34.8%). A Y-graft configuration was found significantly more often in patients with symptomatic early CGF (36.4% vs 16.2%; *p* = 0.035).

**Table 3:** Intraoperative parameters

	Overall (*N* = 92)	No early graft failure (*n* = 37)	Early graft failure (*n* = 55)	*p*-value
*Graft composition*				0.398
Arterial, *n* (%)	35 (38.0)	17 (45.9)	18 (32.7)	
Venous, *n* (%)	7 (7.6)	3 (8.1)	4 (7.3)	
Arterial + venous, *n* (%)	50 (54.3)	17 (45.9)	33 (60.0)	
*Graft form*				**0.035**
– Y-graft, *n* (%)	26 (28.3)	6 (16.2)	20 (36.4)	
*Other procedures, n (%)*	32 (34.8)	11 (29.7)	21 (38.2)	0.404
– MVR, *n* (%)	4 (4.3)	3 (8.1)	1 (1.8)	0.299
– MVP, *n* (%)	14 (15.2)	5 (13.5)	9 (16.4)	0.709
– TVP, *n* (%)	3 (3.3)	0	3 (5.5)	0.271
– AVR, *n* (%)	12 (13.0)	3 (8.1)	9 (16.4)	0.349
– Aortic arch, *n* (%)	3 (3.3)	2 (5.4)	1 (1.8)	0.562
– VSD closure, *n* (%)	1 (1.1)	0	1 (1.8)	1
– LAAA, *n* (%)	4 (4.3)	1 (2.7)	3 (5.5)	0.646
– AF ablation, *n* (%)	7 (7.6)	2 (5.4)	5 (9.1)	0.698
– Endoventricular patch plasty, *n* (%)	1 (1.1)	0	1 (1.8)	1
ECC time (min)	154.8 ± 74.1	147.5 ± 87.6	159.7 ± 63.9	0.470
Cross-clamp time (min)	107.0 ± 55.0	102.0 ± 64.7	110.4 ± 47.8	0.476
Off-pump CABG, *n* (%)	3 (3.3)	1 (2.7)	2 (3.6)	1
*Direct post-surgical support, n (%)*	66 (71.7)	25 (67.6)	41 (74.5)	0.294
– Vasopressive, *n* (%)	61 (66.3)	24 (64.9)	37 (67.3)	0.811
– Inotropic, *n* (%)	26 (28.3)	13 (35.1)	13 (23.6)	0.230
– Mechanical, *n* (%)	4 (4.3)	3 (8.1)	1 (1.8)	0.299

*AF* atrial fibrillation, *AVR* aortic valve replacement, *CABG* coronary artery bypass graft, *ECC* extracorporeal circulation, *LAAA* left atrial appendage amputation, *LIMA* left internal mammary artery, *MVP* mitral valve plasty, *MVR* mitral valve replacement, *RIMA* right internal mammary artery, *TVP* tricuspid valve plasty, *VSD* ventricular septum defect

### Postoperative parameters

Potential indications for CAG included new ECG abnormalities (*n* = 78, 84.8%), echocardiographic changes (*n* = 26, 28.3%), biomarker elevation (*n* = 81, 88%) and prolonged inotropic, vasopressive and/or mechanical support (*n* = 40, 43.5%) (Tab. 4). There were no significant differences between patients with and without symptomatic early CGF in overall ECG abnormalities (*p* = 0.103), ischaemic changes (*p* = 0.196), rhythm disorders (*p* = 0.389) and conductions disorders (*p* = 0.514). Postoperative TTEs were performed in an almost equal number of cases (48.6% vs 47.3%; *p* = 0.897) with a similar rate of new RWMA (27.0% vs 29.1%; *p* = 0.691). In addition, cardiac biomarker elevation was comparable (86.5% vs 89.1%; *p* = 0.706), with no significant differences in median peak TnT, peak CK, ∆TnT or ∆CK (Tab. 4). Haemodynamic status 12 h post-CABG was similar, with prolonged support observed in 40.5% versus 45.5% of patients (*p* = 0.641).

**Table 4:** Postoperative parameters and interventions

	Overall (*N* = 92)	No early graft failure (*n* = 37)	Early graft failure (*n* = 55)	*p*-value
*Abnormal ECG, n (%)*	78 (84.8)	28 (75.7)	50 (90.9)	0.103
– Ischaemic, *n* (%)	71 (77.2)	26 (70.3)	45 (81.8)	0.196
a. ST-segment elevation, *n* (%)	54 (58.7)	20 (54.1)	34 (61.8)	0.458
b. ST-segment depression, *n *(%)	10 (10.9)	2 (5.4)	8 (14.5)	0.306
c. Abnormal repolarisation, *n* (%)	8 (8.7)	1 (2.7)	7 (12.7)	0.137
d. T-wave inversion, *n* (%)	24 (26.1)	7 (18.9)	17 (30.9)	0.199
– Rhythm disorder, *n* (%)	19 (20.7)	6 (16.2)	13 (23.6)	0.389
– Conduction disorder, *n* (%)	24 (26.1)	11 (29.7)	13 (23.6)	0.514
*Laboratory results, n (%)*	81 (88.0)	32 (86.5)	49 (89.1)	0.706
– TnT (12 h post), μg/l	1821 (860–4463)	1746 (768–4807)	1926 (907–4425)	0.805
– CK (12 h post), U/l	1397 (824–2166)	1237 (732–2128)	1495 (979–2230)	0.427
– Peak TnT, μg/l	2766 (1452–6941)	1990 (1395–6380)	2850 (1460–7037)	0.522
– Peak CK, U/l	1627 (1006–3047)	1483 (795–3348)	1776 (1162–3012)	0.602
– ∆TnT, μg/l	1006 (88–2529)	966 (99–2705)	1020 (58–2416)	0.914
– ∆CK, U/l	567 (126–1017)	453 (134–934)	609 (125–609)	0.548
*Haemodynamic support, n (%)*	40 (43.5)	15 (40.5)	25 (45.5)	0.641
– Vasopressive, *n* (%)	37 (40.2)	14 (37.8)	23 (41.8)	0.703
– Inotropic, *n* (%)	26 (28.3)	7 (18.9)	19 (34.5)	0.103
– Mechanical support, *n* (%)	11 (12)	5 (13.5)	6 (10.9)	0.960
*Echocardiography performed, n (%)*	44 (47.8)	18 (48.6)	26 (47.3)	0.897
– New RWMA, *n* (%)	26 (28.3)	10 (27.0)	16 (29.1)	0.691
Hours between CABG and CAG	41.5 ± 63.4	50.0 ± 70.0	35.9 ± 58.6	0.104
*Antithrombotic therapy*				
– DAPT, *n* (%)	50 (54.3)	19 (51.4)	31 (56.4)	0.636
– LMWH, *n* (%)	33 (35.9)	14 (37.8)	19 (34.5)	0.747
– Vitamin K antagonists, *n* (%)	27 (29.3)	11 (29.7)	16 (29.1)	0.947

*CABG* coronary artery bypass graft surgery, *CAG* coronary angiography, *CK* creatinine kinase, *DAPT* dual antiplatelet therapy, *ECG* electrocardiography, *LMWH* low-molecular-weight heparin, *RWMA* regional wall motion abnormalities, *TnT* troponin T

### In-hospital revascularisation

At the time of in-hospital recatheterisation, 42 (45.7%) patients underwent revascularisation with a total of 47 interventions performed (18.9% without early CGF vs 63.6% with early CGF) (Table S1, Electronic Supplementary Material). Overall, PCI was the preferred treatment option (*n* = 38, 80.9%). In subgroup comparisons, PCI was used particularly in bypass (*n* = 10, 55.6%) or anastomotic stenosis cases, including distal (*n* = 15, 55.6%) and Y‑graft anastomoses (*n* = 2, 66.7%). Re-CABG was used mainly in more complex cases like bypass kinking (*n* = 5, 41.7%). Conservative management was applied in cases with limited revascularisation options or milder failures such as reduced bypass flow (*n* = 4, 54.1%) (Tab. 1).

### Follow-up and multivariate analysis

**Fig. 2:**
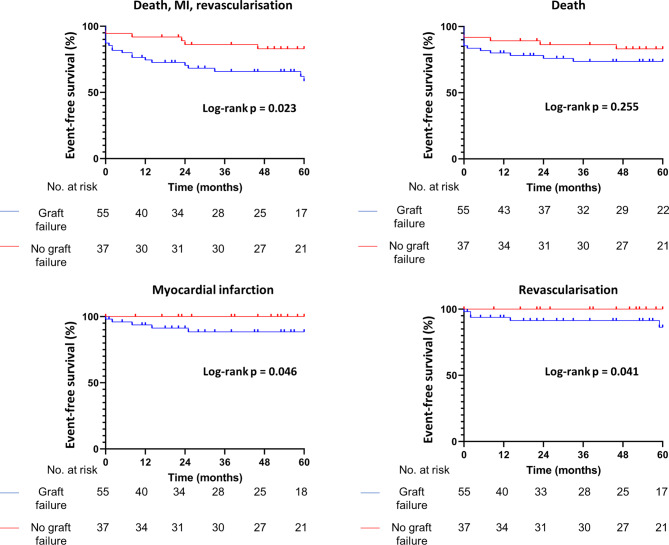
Event-free follow-up. Event-free survival of patients with (*blue line*) and without (*red line*) symptomatic early coronary graft failure. *MI* myocardial infarction

The median follow-up after in-hospital recatheterisation was 33 (11–60) months for patients with symptomatic early CGF and 60 months (38–60) for patients without symptomatic early CGF (*p* = 0.005). Five-year follow-up data were available in 54 patients (58.7%), with MACE observed in 26 (28.3%), of whom 20 (76.9%) had early CGF. Patients with symptomatic early CGF showed a significantly lower MACE-free survival rate over a median follow-up period of 33 months (*p* = 0.023), although all-cause mortality did not differ significantly (*p* = 0.255) (Fig. 2). Multivariate analysis showed that venous graft integration (*p* = 0.005), Y‑graft configuration (*p* = 0.002) and prolonged inotropic use (*p* = 0.032) were significantly associated with symptomatic early CGF (Table S2, Electronic Supplementary Material).

## Discussion

The study defines symptomatic early CGF angiographically and identifies clinical and perioperative risk factors potentially associated with its occurrence. The main findings are: (1) early CGF was observed in the majority of post-CABG patients undergoing clinically indicated CAG before discharge; (2) MACE rates were higher in symptomatic early CGF patients over a median follow-up period of 33 months; (3) venous graft integration, Y‑graft configuration and prolonged inotropic support were associated with symptomatic early CGF. However, these results should be interpreted with caution due to the intrinsic limitations of the study.

Most patients (60%) undergoing post-CABG CAG exhibited symptomatic early CGF. These findings are consistent with those of previous studies, which reported an incidence of CGF ranging from 39 to 78% [5–7, 10, 12, 13]. This wide range might be attributed to the differences in inclusion criteria, such as concomitant surgery and CAG performance in unstable patients. Our study significantly extends the scope of previous research by incorporating an evaluation over a longer follow-up period, providing deeper insights into long-term outcomes.

The majority of patients with symptomatic early CGF (63.6%) underwent in-hospital revascularisation. Performing CAG in patients suspected of PMI based on the indicators in this study, therefore, appears warranted. However, despite interventions, symptomatic early CGF patients had an increased risk of MACE (Fig. 2), likely because CABG patients need fewer revascularisations than patients treated with PCI [14]. Therefore, future studies should focus on strategies to improve outcomes in patients with early CGF.

Identifying patients at risk of early CGF requiring CAG remains challenging. Asymptomatic early CGF may occur, and while traditional ischaemia indicators were common and guided CAG decisions, they were not associated with symptomatic early CGF (Table S2, Electronic Supplementary Material). For instance, cardiac biomarker levels in this study (Tab. 4) were high, guiding CAG performance, but were not associated with symptomatic early CGF. These findings align with the observations of Weidenmann et al., suggesting multiple indicators are needed to indicate the presence of PMI [15]. Although our study, within this specific population, did not identify a statistically significant association to support this conclusion, the results suggest the potential value of conducting a prospective study comparing post-CABG patients with early CGF with patients not undergoing in-hospital CAG.

Venous graft integration, Y‑graft configuration and prolonged inotropic support, on the other hand, were associated with symptomatic early CGF and could be incorporated in clinical decision making. Previous research has shown that venous grafts have the highest failure rates (up to 40–50%) [16]. In this study, particularly distal anastomoses of Y‑grafts, revascularising predominantly the anterolateral territory, were at risk. Y‑graft failure might lead to inadequate perfusion of a large part of the myocardium, impairing haemodynamic stability. Thus, when early CGF is suspected post-CABG, assessing the inotropic need 12 h after surgery and examining the anterolateral territory using echocardiography could be beneficial.

Establishing specific distinct threshold values for cardiac biomarkers, considering intraoperative factors like concomitant procedures and cross-clamp duration, may be useful since TnT levels rise in all postoperative patients due to non-graft-related myocardial injury [17]. Additionally, a single elevated cardiac biomarker reading should not be interpreted as a sign of PMI, as a trend of increasing TnT levels offers more significant prognostic insights [18].

Following the results of this study, future studies should prioritise investigating composite indicators of ischaemia, comparing patients with early CGF to CABG patients without a clinical indication for the performance of CAG. Additionally, the relationship between graft failure type, the territory supplied and clinical outcomes should be evaluated.

### Limitations

Several limitations should be acknowledged, primarily due to the modest sample size from a single centre and the retrospective nature of the study. This is particularly relevant when interpreting the clinical outcomes observed. Secondly, we did not analyse CABG patients who did not require CAG, so the results apply only to symptomatic patients. The timing of graft failure mechanisms was difficult to determine due to delays between occurrence and detection through CAG. Lastly, treatment protocols may have varied during the inclusion period, potentially influencing the occurrence of graft failure. The conclusions of this study should therefore be interpreted as hypothesis generating.

## Conclusions

Symptomatic early CGF was observed in the majority of post-CABG patients undergoing clinically indicated CAG prior to discharge. Patients with symptomatic early CGF exhibited higher MACE rates over a median follow-up period of 33 months. Venous graft integration, Y‑graft configuration and prolonged use of inotropic agents were associated with symptomatic early CGF. However, the clinical findings should be interpreted with caution.

## Supplementary Information

The supplementary materials provide additional data and analyses to support the findings discussed in the main text:

Table S1: Data on interventions following in-hospital coronary angiography, comparing patients with and without symptomatic early coronary graft failure.

Table S2: Data on potential predictors of graft failure.
