# Supplementary material for: Symptomatic early coronary graft failure in bypass surgery patients: incidence, predictors and clinical impact
Source: Neth Heart J. 2025 Jan 20;33(3):93–102. doi: 10.1007/s12471-024-01926-z (PMC11845632; doi:10.1007/s12471-024-01926-z)
Supplement: Supplementary file 1 — Table S1: Data on interventions following in-hospital coronary angiography, comparing patients with and without symptomatic early coronary graft failure. [file 12471_2024_1926_MOESM1_ESM.docx]

**Table S1** Treatment After In-Hospital Coronary Angiography in Patients With and Without Symptomatic Early Coronary Graft Failure

|  | **Overall**  **N=92** | **No early Graft Failure**  **N=37** | **Early Graft failure**  **N=55** |
| --- | --- | --- | --- |
| **Intervention performed, n(%)** | 42(45.7) | 7(18.9) | 35(63.6) |
| PCI bypass, n(%) | 7(7.6) | 0 | 7(12.7) |
| PCI native vessel, n(%) | 31(33.7) | 7(18.9) | 24(43.6) |
| Re-CABG, n(%) | 9(9.8) | 0 | 9(16.4) |
| **Conservative treatment, n(%)** | 50(54.3) | 30(81.1) | 20(36.4) |

CABG= coronary artery bypass graft; PCI= percutaneous coronary intervention.
