# Supplementary material for: Symptomatic early coronary graft failure in bypass surgery patients: incidence, predictors and clinical impact
Source: Neth Heart J. 2025 Jan 20;33(3):93–102. doi: 10.1007/s12471-024-01926-z (PMC11845632; doi:10.1007/s12471-024-01926-z)
Supplement: Supplementary file 2 — Table S2: Data on potential predictors of graft failure. [file 12471_2024_1926_MOESM2_ESM.docx]

**Table S2** Uni- and multivariate logistic regression analyses of the variables associated with early coronary graft failure

| **Variable** | **Univariable analysis** | | |  | **Multivariable analysis** | | |  |
| --- | --- | --- | --- | --- | --- | --- | --- | --- |
|  | **Odds ratio** | **95% confidence interval** | **P-value** |  | **Odds**  **ratio** | **95% confidence interval** | **P-value** |  |
| Age | 1.009 | 0.967-1.052 | 0.688 |  |  |  |  |  |
| Gender (male) | 1.599 | 0.547-4.676 | 0.391 |  |  |  |  |  |
| Hypertension | 1.193 | 0.507-2.808 | 0.686 |  |  |  |  |  |
| Diabetes | 1.108 | 0.437-2.808 | 0.829 |  |  |  |  |  |
| Hypercholesterolemia | 0.966 | 0.536-1.741 | 0.909 |  |  |  |  |  |
| History of smoking | 1.134 | 0.492-2.615 | 0.767 |  |  |  |  |  |
| Preoperative LV function | 1.104 | 0.756-1.613 | 0.607 |  |  |  |  |  |
| BMI | 1.107 | 0.994-1.233 | 0.064 |  | 1.113 | 0.990-1.251 | 0.072 |  |
| Elevated cardiac biomarkers | 1.276 | 0.359-4.533 | 0.706 |  |  |  |  |  |
| Venous graft integration | 1.355 | 0.817-2.107 | 0.178 |  | 2.543 | 1.330-4.862 | **0.005** |  |
| Graft form (Y) | 2.952 | 1.051-8.290 | **0.040** |  | 9.757 | 2.342-40.644 | **0.002** |  |
| Support 12-hours after surgery (vasopression) | 1.181 | 0.503-2.772 | 0.703 |  |  |  |  |  |
| Support 12-hours after surgery (inotropics) | 2.262 | 0.838-6.105 | 0.107 |  | 3.505 | 1.111-11.053 | **0.032** |  |
| Support 12-hours after surgery (mechanical) | 0.784 | 0.221-2.784 | 0.784 |  |  |  |  |  |
| ECG changes | 2.055 | 0.658-6.416 | 0.215 |  |  |  |  |  |
| Echo performed | 0.946 | 0.411-2.179 | 0.897 |  |  |  |  |  |
| Echo RWMA | 1.280 | 0.378-4.336 | 0.692 |  |  |  |  |  |

^BMI=body mass index; CK=creatinine kinase; ECG=electrocardiogrpahy; RWMA=regional wall motion abnormalities.^
